# Targeted anti-inflammatory therapeutics in asthma and chronic obstructive lung disease

**DOI:** 10.1016/j.trsl.2015.08.004

**Published:** 2016-01

**Authors:** Andrew L. Durham, Gaetano Caramori, Kian F. Chung, Ian M. Adcock

**Affiliations:** a Airway Diseases Section, National Heart and Lung Institute, Imperial College London, London, UK; b Biomedical Research Unit, Royal Brompton and Harefield NHS Trust, London, UK; c Section of Respiratory Diseases, Centro per lo Studio delle Malattie Infiammatorie Croniche delle Vie Aeree e Patologie Fumo Correlate dell'Apparato Respiratorio (CEMICEF; ex Centro di Ricerca su Asma e BPCO), Sezione di Medicina Interna e Cardiorespiratoria, Università di Ferrara, Ferrara, Italy

**Keywords:** AHR, airway hyperresponsiveness, ACQ, Asthma Control Questionnaire, ACOS, asthma-COPD overlap syndrome, BAL, bronchoalveolar lavage, CLCA1, chloride channel regulator 1, COPD, chronic obstructive lung disease, CS, corticosteroids, CXCR, CXC chemokine receptor, EGF, epidermal growth factor, EGFR, epidermal growth factor receptor, FKBP51, FK506-binding protein 51, FP, fluticasone propionate, FEV_1_, Forced expiratory volume in 1 second, FeNO, fraction of exhaled nitric oxide, GR, glucocorticoid receptor, GM-CSF, Granulocyte-macrophage colony-stimulating factor, HDACs, histone deacetylases, HNE, Human neutrophil elastase, IgE, Immunoglobulin E, ICS, inhaled corticosteroids, LABAs, Long-acting beta-adrenoceptor agonists, mRNA, messenger RNA, MAbs, monoclonal antibodies, PDE, phosphodiesterase, PI3K, phosphoinositide-3-kinase, RT-qPCR, Real time quantative polymerase chain reaction, SAL, salmeterol, SERPINB2, serpin peptidase inhibitor, clade B, member 2, sIL-4R, soluble IL-4 receptor, GOLD, The Global Initiative for Chronic Obstructive Lung Disease, TSLP, Thymic stromal lymphopoietin, TORCH, Towards a Revolution in COPD Health

## Abstract

Asthma and chronic obstructive pulmonary disease (COPD) are chronic inflammatory diseases of the airway, although the drivers and site of the inflammation differ between diseases. Asthmatics with a neutrophilic airway inflammation are associated with a poor response to corticosteroids, whereas asthmatics with eosinophilic inflammation respond better to corticosteroids. Biologicals targeting the Th2-eosinophil nexus such as anti–interleukin (IL)-4, anti–IL-5, and anti–IL-13 are ineffective in asthma as a whole but are more effective if patients are selected using cellular (eg, eosinophils) or molecular (eg, periostin) biomarkers. This highlights the key role of individual inflammatory mediators in driving the inflammatory response and for accurate disease phenotyping to allow greater understanding of disease and development of patient-oriented antiasthma therapies. In contrast to asthmatic patients, corticosteroids are relatively ineffective in COPD patients. Despite stratification of COPD patients, the results of targeted therapy have proved disappointing with the exception of recent studies using CXC chemokine receptor (CXCR)2 antagonists. Currently, several other novel mediator-targeted drugs are undergoing clinical trials. As with asthma specifically targeted treatments may be of most benefit in specific COPD patient endotypes. The use of novel inflammatory mediator-targeted therapeutic agents in selected patients with asthma or COPD and the detection of markers of responsiveness or nonresponsiveness will allow a link between clinical phenotypes and pathophysiological mechanisms to be delineated reaching the goal of endotyping patients.

## Introduction

Asthma and chronic obstructive pulmonary disease (COPD) affect more than 500 million people worldwide representing the 2 most common chronic inflammatory diseases of the lower airways.^1^, ^2^ This review summarizes some of the recent evidence indicating how the use of therapeutics targeting specific inflammatory mediators has indicated their role in disease pathophysiology and also highlighted the importance of subphenotyping these diseases to optimize the response to these targeted drugs.

The current consensus definition of asthma is “an (sic) heterogeneous disease, usually characterized by chronic airway inflammation. It is defined by the history of respiratory symptoms such as wheeze, shortness of breath, chest tightness and cough that vary over time and in intensity, together with variable airflow obstruction.”^1^ Patients with asthma have variable airflow obstruction and airway hyper-responsiveness (AHR).^3^ Asthma affects 10%–12% of the adult population in Europe and most high (€20.65 billion) annual costs of asthma in Europe are because of patients with severe disease who do not respond well to conventional anti-inflammatory corticosteroids that is the mainstay treatment of mild-moderate asthma.^4^

The analysis of airway biopsies after bronchoscopy and the introduction of induced sputum analysis allowed the inflammatory nature of the asthmatic airways to be confirmed.^5^, ^6^, ^7^ These analyses revealed the presence of eosinophils and Th2 cytokines particularly interleukin (IL)-2 and IL-4,^8^ which emphasized the Th2-driven nature of asthmatic inflammation. As a result asthma was, for a long time, considered as a single Th2-driven eosinophilic disease whose diagnosis is based on the patient presenting with an intermittent wheeze, dyspnea, and cough. However, it was clear that the presentation and natural history of the disease differ between patients; some asthmatics undergo clinical remission during adolescence, some patients have more severe disease, some asthmatics are nonallergic or atopic, whereas others have exercise-induced asthma.^9^, ^10^

Later studies showed that although eosinophils were present in many asthmatic biopsies, some subjects, particularly those with more severe disease, also demonstrated increased levels of neutrophils.^11^ Similarly, Gibson et al have shown different types of sputum cellular composition in asthma with some subjects having predominant eosinophilia, others more neutrophilic or with a mixed cell composition, and another group with paucicellular sputum.^12^, ^13^ This has led to the idea of asthma being a complex disease or even a group of “diseases” caused by different pathophysiological mechanisms.^9^, ^10^

The Global Initiative for Chronic Obstructive Lung Disease guidelines define COPD as “a common preventable and treatable disease, characterized by persistent airflow limitation that is usually progressive and associated with an enhanced chronic inflammatory response in the airways and the lung to noxious particles or gases. Exacerbations and comorbidities contribute to the overall severity in individual patients.”^14^ COPD is expected to rise from the 4th to the 3rd leading cause of morbidity and mortality worldwide within the next 5 years.^14^ According to the World Health Organization, approximately 3 million people in the world die as a consequence of COPD every year.^15^ The estimated annual costs of COPD are $24 billion and 70% are related to exacerbations requiring hospitalization.^2^ In developed countries, the leading risk factor for COPD is cigarette smoking with smokers constituting more than 90% of COPD patients.^14^ In less-well developed countries, biomass fuel used in cooking and other environmental pollutants are major factors.^16^, ^17^

The pathologic features of COPD are lung parenchymal destruction (pulmonary emphysema), inflammation of the small (peripheral) airways (respiratory bronchiolitis), and inflammation of the central airways. Inflammation occurs within all these compartments (central and peripheral airways and lung parenchyma).^18^ The major sites of airflow obstruction are the small airways and lung parenchyma in COPD.^19^, ^20^

## Corticosteroid Responsiveness in Asthma

Asthma was implicated as a chronic inflammatory disease, and this was confirmed when the potent anti-inflammatory prednisolone was shown to be of benefit in patients with asthma. Some of the original studies with oral prednisolone highlighted that blood levels of eosinophils were not altered in patients who were more refractory to treatment. This provided early evidence for the concept of relatively corticosteroid-resistant asthma.^21^ However, the introduction of inhaled corticosteroids (ICSs) resulted in such a dramatic improvement in the asthma symptoms of most asthmatics^22^, ^23^ that these earlier studies indicating that only 40% of asthmatics responded well, at least in terms of an improvement in forced expiratory volume in 1 second (FEV_1)_, was ignored. In common with most chronic inflammatory diseases, corticosteroids are able to attenuate all measures of inflammation in asthmatic airways leading to a reversal in the decline in FEV_1_ associated with the disease and a reduction in AHR back to normal levels. However, ICSs do not cure asthmatics as many of the symptoms of asthma and the inflammation return on their discontinuation.^24^, ^25^

Asthmatic patients who respond poorly to ICSs or even oral corticosteroids, termed severe asthmatics, often have more frequent exacerbations requiring hospitalization and depression associated with the chronic nature of the disease, which is not controlled by conventional therapy.^9^ There is an urgent need for new effective anti-inflammatory drugs that could treat or even ultimately cure these patients as they will have a profound effect on an individual patient's welfare and also on the huge associated societal costs.^24^ Relative corticosteroid insensitivity is seen in a subset of patients across all chronic inflammatory diseases with a commensurate increase in healthcare and societal costs.^26^

Asthma is now recognized as a syndrome with many potential phenotypes and endotypes and understanding these is essential in determining the most effective therapeutic regimen suitable for each patient.^10^ Several large consortia in Europe and the USA, as well as individual research centers, have been instrumental in defining subgroups of asthma and severe asthma based on clinical features.^10^, ^27^, ^28^ For example, 5 clusters of asthma were identified by SARP^29^ and 4 clusters from Leicester.^30^ In both studies, severe asthmatics were distributed among several clusters. The therapeutic efficacy of some drugs was enhanced by taking cytopathologic and histopathologic inflammatory characteristics and or genomic signatures into account. For instance, neutrophilic airway inflammation was associated with poor corticosteroid responses^31^ and corticosteroid treatment guided by eosinophilic inflammation led to better disease outcome than standard clinical management.^32^, ^33^ Interestingly, patient selection guided by sputum eosinophils also appears to be a better predictor of a patient's responses to anti–IL-5 antibody treatment.^34^, ^35^, ^36^

## Inhibiting Inflammatory Mediators in Asthma

More than 100 mediators have now been implicated in asthmatic inflammation, including multiple cytokines, chemokines, and growth factors.^24^ Blocking a single mediator is therefore unlikely to be very effective in this complex disease, and mediator antagonists have so far not proved to be very effective compared with drugs that have a broad spectrum of anti-inflammatory effects, such as corticosteroids. However, blocking Th2 cytokines such as IL-4, IL-5, and IL-13 despite not been completely explored has shown some promising results in selected patients with asthma.

## IL-5-Targeted Therapy in Asthma

The Th2 cell cytokine, IL-5, plays an important role in eosinophil maturation, differentiation, recruitment, and survival. IL-5 knockout mice appeared to confirm a role in asthma models in which eosinophilia and AHR are markedly suppressed. The humanized anti–IL-5 monoclonal antibodies (MAbs), mepolizumab (formerly termed SCH55700) and reslizumab (formerly Res-5-0010), have been developed for clinical use.^37^ Another fully humanized MAb benralizumab (previously named MEDI-563) instead targets eosinophils by binding IL-5 receptor α (IL-5Rα) inhibiting IL-5 binding and inducing eosinophil apoptosis through antibody-dependent cell-mediated cytotoxicity.^38^

It has been demonstrated in earlier studies that using mepolizumab in mild-moderate asthmatics was very effective at reducing sputum and blood eosinophils but had no effect on clinical signs and symptoms suggesting that the concept of asthma as a Th2-driven eosinophilic disease was incorrect.^39^ Subsequent studies showed that the same dose of mepolizumab only partially reduced eosinophil numbers within bronchial biopsies of asthmatics, which may have accounted for the persistence of symptoms.^40^ However, this approach was unable to distinguish potential responders from nonresponders.

On the basis of earlier work showing a cluster of patients with severe asthma had inappropriate levels of sputum eosinophils, Haldar et al examined whether reducing eosinophilia in these patients would have any effect on the clinical features of asthma particularly exacerbations.^41^ Sixty-one subjects who had refractory eosinophilic asthma and a history of recurrent severe exacerbations received intravenous infusions of 750 mg mepolizumab monthly for 1 year in a randomized, double-blind, placebo-controlled, parallel-group study (Current Controlled Trials number, ISRCTN75169762.). Mepolizumab significantly lowered eosinophil counts in the blood (*P* < 0.001) and sputum (*P* = 0.002), and this was associated with a reduction in exacerbations (2.0 vs 3.4 mean exacerbations per subject) compared with placebo. There were no significant differences between the groups with respect to symptoms, FEV_1_ after bronchodilator use, or AHR.^41^

An unblinded follow-up analysis of 56 of the original 61 subjects for 12 months indicated that withdrawal of mepolizumab resulted in an increase in blood and sputum eosinophils that preceded the rise in exacerbations. Twelve months after stopping mepolizumab, exacerbation frequency was similar in the placebo and mepolizumab groups. Not unexpectedly, no effects on FEV_1_ or fraction of exhaled nitric oxide were seen after cessation of mepolizumab therapy. This supports the concept that these events are related and have pathophysiological importance.^42^

These results were replicated in a follow-up multicenter, double-blind, placebo-controlled study (ClinicalTrials.gov number, NCT01000506) in 621 patients with similar characteristics as the original study. There was a 52% reduction in clinically significant exacerbations with 75 mg mepolizumab, and blood eosinophils appeared to be the best predictor of response.^35^

Furthermore, similar results were observed whether mepolizumab was delivered intravenously (75 mg) or subcutaneously (100 mg) for 32 weeks with a 47% and 53% reduction in exacerbations, respectively. This study (ClinicalTrials.gov number, NCT01691521) also reported an increase in FEV_1_ in all patients receiving mepolizumab of approximately 100 mL and a resultant improvement in asthma quality of life and asthma control questionnaire scores.^36^ One hundred milligrams of mepolizumab given subcutaneously had a significant glucocorticoid-sparing effect, reduced exacerbations, and improved control of asthma symptoms in patients requiring daily oral glucocorticoid therapy to maintain asthma control (ClinicalTrials.gov number, NCT01691508).^43^

These latter 2 important new studies suggest that the regular monthly treatment with subcutaneous injections of mepolizumab reduce exacerbation rates by ∼50% in asthmatic patients with persistent peripheral blood eosinophilia and persistent symptoms despite high-dose ICS and additional controller therapy, and frequent exacerbations. Mepolizumab is also able to reduce oral steroid requirement by ∼50% in similar asthmatic patients who additionally required oral prednisone to control their asthma.^36^, ^43^, ^44^

Two other anti–IL-5R MAbs, benralizumab and reslizumab, had effects similar to mepolizumab with respect to decreasing rates in asthmatic patients with poorly controlled disease and high levels of blood and/or sputum eosinophils despite high doses of ICS therapy. The anti–IL-5Rα MAb benralizumab delivered either intravenously or subcutaneously reduced eosinophil counts in airway mucosa and submucosa, sputum, bone marrow, and peripheral blood.^38^ In a large controlled clinical trial, benralizumab only reduced exacerbation rates compared with placebo (57% vs 43%) in the subgroup of patients with persistent asthma and high baseline blood eosinophils.^45^ There are currently many ongoing controlled clinical trials investigating the efficacy and safety of the addition of benralizumab to the standard of care in the treatment of adult asthmatic patients with different levels of asthma severity (NCT02075255, NCT02322775, NCT01928771, and NCT02258542).

Reslizumab reduces sputum eosinophils in patients with eosinophilic asthma that is poorly controlled with high-dose ICSs compared with placebo.^46^ In 2 studies, patients whose asthma was inadequately controlled by medium-to-high doses of ICS-based therapy and who had blood eosinophils >400 cells/μL and one or more exacerbations in the previous year, intravenous reslizumab had a significant reduction in the frequency of asthma exacerbations compared with those receiving placebo.^47^

## IL-13-Targeted Therapy in Asthma

As part of the Th2 hypothesis of asthma, IL-13 has been shown to play a critical role in various aspects of airway inflammation and epithelial remodeling including goblet cell metaplasia and epithelial-mesenchymal signaling. This leads to increased subepithelial fibrosis or airway smooth muscle hyperplasia.^48^ Blocking IL-13, but not IL-4, in animal models of asthma prevents the development of AHR after allergen, despite a strong eosinophilic response. In addition, soluble IL-13Rα2 is effective in blocking the actions of IL-13, including immunoglobulin E (IgE) production, pulmonary eosinophilia, and AHR in animal models of asthma.^37^

Airway epithelial cells are a major target for corticosteroids and their effectiveness in asthma may relate to their ability to modulate the production of inflammatory mediators from these cells, which have downstream effects on other inflammatory cells. In an attempt to address this issue, Woodruff et al collected epithelial cells obtained by bronchial brushings from asthmatic subjects undergoing a randomized controlled trial of ICSs and from healthy subjects.^49^

Transcriptomic analysis of these cells identified 2 distinct groups termed Th2-high and Th2-low based on the differential expression of IL-13–inducible genes (periostin, chloride channel regulator 1, and serpin peptidase inhibitor, clade B, member 2)—a Th2-high signature. Corticosteroid treatment downregulated the expression of these 3 genes and markedly upregulated the expression of FK506-binding protein 51. High basal expression of this Th2-high signature was associated with a good clinical response to corticosteroids, whereas high FK506-binding protein 51 expression was linked to a poor clinical response. These results were confirmed in primary epithelial cell cultures. Overall, the Th2-high group was associated with sputum eosinophilia and AHR and consistent with the predictive value of sputum eosinophilia, the Th2-high phenotype responded clinically to corticosteroids (CS) resulting in reduced expression of these Th2 biomarkers.^49^, ^50^

Measurement of the Th2 signature in bronchial epithelium depends on invasive airway sampling and hence noninvasive biomarkers of this phenotype are desirable. Sputum is a more accessible compartment to determine airway Th2-high vs Th2-low status. Real time quantative polymerase chain reaction (RT-qPCR) analysis of sputum cell pellets from 37 asthmatic patients and 15 healthy control subjects demonstrated that the gene expression levels of chloride channel regulator 1 and periostin, but not serpin peptidase inhibitor, clade B, member 2, were significantly higher than normal in sputum cells from asthmatic subjects.^51^ However, the composite expression of IL-4, IL-5, and IL-13 messenger RNA was able to demonstrate Th2-high status in these patients. These were characterized by more severe measures of asthma and increased blood and sputum eosinophilia. This Th2-gene mean value was stable at the upper and lower limits but at intermediate levels raising issues about the utility of this signal for these patients.^51^ The Th2-high signature is a variable being correlated with local allergic signals and eosinophilia^52^ and has also been linked to markers of airway remodeling.^50^

A comparison of IgE levels, blood eosinophil numbers, fraction of exhaled nitric oxide levels, and serum periostin levels in 59 patients with severe asthma indicated that serum periostin level was the single best predictor of airway eosinophilia (*P* = 0.007).^53^ It made sense, therefore, to use periostin levels as a biomarker for anti–IL-13 therapy particularly because some patients with uncontrolled asthma continue to have increased levels of IL-13 in the sputum, despite the use of systemic corticosteroids and ICSs.^54^

Lebrikizumab, an MAb to IL-13, improved lung function in a randomized, double-blind, placebo-controlled study of 219 adults who had asthma that was inadequately controlled despite inhaled glucocorticoid therapy (ClinicalTrials.gov number, NCT00930163).^55^ Overall, the patients on lebrikizumab had a 5.5% increase in FEV_1_ than those in the placebo group. When patients were divided according to high or low serum periostin levels, those with high periostin had greater improvement in lung function with lebrikizumab (8.2%) than those with low periostin levels (1.6%). Patient selection, however, is the key to success with these drugs because lebrikizumab failed to increase FEV_1_ in 212 adult asthmatics not receiving ICSs irrespective of periostin levels.^56^

Similarly, subcutaneous tralokinumab (formerly CAT-354), an IL-13–neutralizing IgG4 MAb, improves lung function but not global asthma control scores in adults with moderate-to-severe uncontrolled asthma despite controller therapies.^57^ There is an ongoing large controlled clinical trial investigating the efficacy of tralokinumab in adults and adolescents with oral corticosteroid-dependent asthma (https://clinicaltrials.gov/ct2/show/NCT02281357).

## IL-4-Pathway-Targeted Therapy in Persistent Asthma

IL-4 analogs that act as antagonists have been developed, which fail to induce signal transduction and block IL-4 effects in vitro. These IL-4 antagonists prevent the development of asthma in vivo in animal models.^37^ However, the development of pascolizumab (SB 240683), a humanized anti–IL-4 antibody,^58^ and a blocking variant of human IL-4 (BAY36-1677) has apparently been discontinued.^37^

A soluble IL-4 receptor (sIL-4R) that acts as an IL-4R antagonist and for this reason is a dual IL-4/IL-13 pathway antagonist, termed pitrakinra, has also been developed. A single nebulized dose of sIL-4R prevents the fall in lung function induced by glucocorticoid withdrawal in moderate to severe asthmatics^59^ and allergen-induced FEV_1_ decrease in atopic asthmatics.^60^ Weekly nebulization of sIL-4R for more than 3 months improves asthma control.^59^ Interestingly amino acid changes in the 3′-end of the IL-4Rα gene or closely proximal variants predict pitrakinra efficacy.^61^ However, further studies in patients with milder asthma proved disappointing and the clinical development of this compound has now been discontinued.^37^

Dupilumab (SAR231893/REGN668) is a fully humanized anti–IL-4Rα MAb, which reduced asthma exacerbations when long-acting beta-adrenoceptor agonists (LABAs) and ICSs were withdrawn from patients with persistent asthma, moderate-to-severe asthma, and who had a blood eosinophil count >300 cells/μL or a sputum eosinophil level >3%. This was associated with improved lung function and reduced levels of Th2-associated inflammatory markers.^62^ In contrast, AMG 317, another fully humanized anti–IL-4Rα MAb, did not demonstrate any clinical efficacy in patients with moderate-to-severe asthma.^63^

## Other Anti-inflammatory Pathways under Clinical Investigation in Persistent Asthma

In a short 4-week double-blind randomized study, MK-7123/SCH527123, a CXC chemokine receptor (CXCR)2 antagonist, reduced sputum neutrophilia by 36% in patients with severe asthma, although it had no effect on lung function or on an asthma quality of life questionnaire.^64^ Longer studies are warranted in these patients.

Despite good results in preclinical studies, brodalumab, an anti–IL-17Rα MAb, had no effect on asthma control questionnaire score, FEV_1_, symptom scores, or number of symptom-free days in 302 subjects with inadequately controlled moderate-severe asthma taking regular ICSs. Subgroup analysis of the high-reversibility group was of uncertain significance and will require a further clinical study (www.clinicaltrials.gov, NCT01199289).^65^ Secukinumab (AIN457), an anti–IL-17 MAb that selectively neutralizes IL-17A, is starting phase II trials in asthmatic subjects who are not adequately controlled with ICSs and long-acting β2-agonists (NCT01478360).

Thymic stromal lymphopoietin (TSLP) is a bronchial epithelial cell–derived cytokine that may be important in initiating allergic inflammation. MEDI9929 (formerly AMG 157) is a human anti-TSLP MAb that binds human TSLP and prevents receptor interaction.^66^ Treatment with AMG 157 reduced allergen-induced bronchoconstriction and blood and sputum before and after allergen challenge.^66^ There is an ongoing clinical trial investigating the efficacy of MEDI9929 in not well-controlled severe persistent asthma (https://www.clinicaltrials.gov/ct2/show/NCT02054130).

## Summary on the Current Approach of Targeting Novel Anti-inflammatory Pathways in Asthma

**Fig 1:**
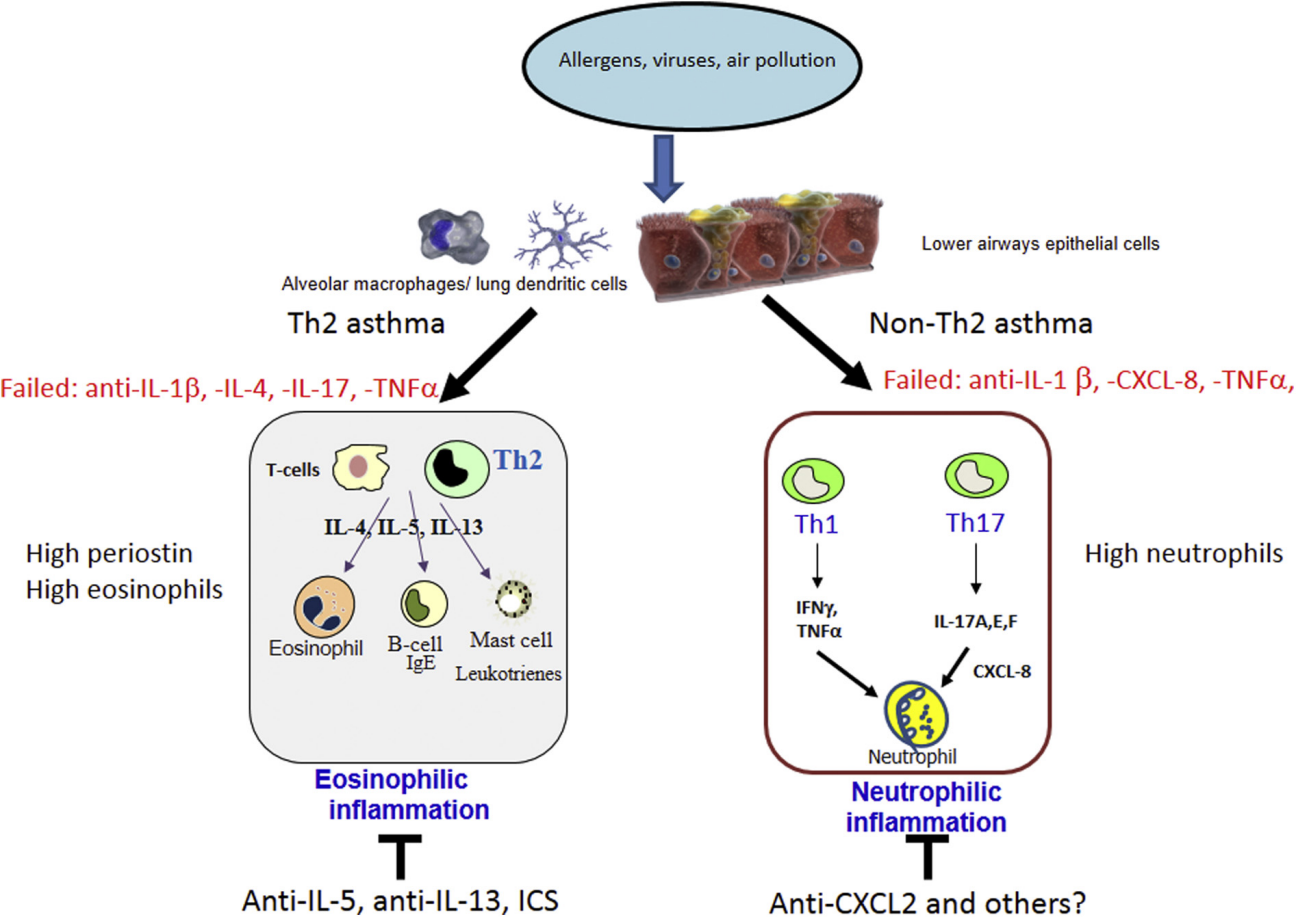
Asthma occurs in the airway because of the combined responses of structural cells such as epithelial cells and immune cells, macrophages and dendritic cells in response to aero allergens, viruses, or other environmental challenges. This results in the production of a host of inflammatory mediators that drive the disease process. There are at least 2 major types of asthma—Th2-high and Th2-low—dependent on the presence of selected Th2 molecular signatures including periostin and high levels of sputum and blood eosinophils in response to Th2 cytokines such as IL-4, IL-5, and IL-13. Asthmatic patients with uncontrolled severe disease despite high doses of ICSs and having high eosinophils and blood periostin respond to anti–IL-5 and anti–IL-13 therapy. There is a negligible clinical response to anti–IL-1, anti–IL-4, anti–IL-17, or TNF-α treatment. Non-Th2 patients are believed to have a more mixed lymphocyte profile involving Th1 and Th2 cells. They respond to anti-CXCR2 antagonists but not to anti–IL-1, anti–IL-8, or TNF-α treatment. Studies using anti–IL-17 or other antineutrophil approaches are yet to be completed. ICS, inhaled corticosteroid; IFN, interferon; IgE, immunoglobulin E; IL, interleukin; TNF, tumor necrosis factor.

These classifications and biomarkers are useful as a start (Fig 1) but are not optimal and do not provide detailed information regarding biomarkers or pathways involved in corticosteroid insensitivity or in Th2-low asthma. In addition, there are still many unresolved issues on the inclusion criteria of the asthmatic patients in these trials. For example, considering the very low compliance to regular antiasthmatic drugs (both by inhaled and oral routes), even in patients with severe persistent asthma.^67^ We eagerly need objective measures of this compliance to avoid to erroneously attribute difficult-to-control asthma to a reduced responsiveness to corticosteroids.^68^ Another key issue is represented by accurate differential diagnosis of patients with severe persistent asthma to avoid the erroneous inclusion in these clinical trials of patients with other diseases such as the Churg-Strauss syndrome, chronic eosinophilic leukemia, lymphocyte-variant hypereosinophilia, and idiopathic hypereosinophilic syndrome that may mimick the clinical features of asthma and are usually associated with minimal response to ICSs but may respond to anti-Th2 cytokine treatment.^69^ Furthermore, the Th2 signatures investigated so far are not very specific and more selective Th2 biomarkers such as the Th2 transcription factor GATA-3 may be more rewarding^70^ and compounds targeting this pathway are under clinical development.^71^

## Chronic Obstructive Pulmonary Disease

The progressive chronic airflow limitation in COPD results from 2 major pathologic processes: remodeling and narrowing of small airways and destruction of the lung parenchyma (pulmonary emphysema) with consequent loss of the alveolar attachments of these airways. This results in diminished lung recoil, higher resistance to flow, and closure of small airways at higher lung volumes during expiration, with consequent air trapping in the lung. This leads to the characteristic lung hyperinflation, which gives rise to the sensation of dyspnea and decreased exercise tolerance. Both small-airway remodeling and narrowing and pulmonary emphysema result from chronic peripheral lung inflammation.^72^

There are increased number of macrophages, neutrophils, T lymphocytes, dendritic cells, and B lymphocytes in the lower airways of patients with stable COPD.^19^ However, the predominant inflammatory cell type varies with disease severity, with an increased number of neutrophils and B lymphocytes in the most severe (III and IV) grades.^19^, ^20^ Eosinophils are more pronounced during viral-induced severe COPD exacerbations.^73^ In contrast to asthma, there is a preferential increase in CD8+ and CD4+ cells associated with the production of interferon gamma,^74^ although Th2 cytokines such as IL-4 are also increased in COPD patients.^75^ Th17 cell numbers are also increased in bronchial biopsies of COPD patients.^76^ The role of the increased number of B lymphocytes in severe and very severe COPD is unknown, but they may be associated with the increased presence of autoantibodies directed against oxidized proteins.^77^

## Inhibiting Inflammatory Mediators in COPD

### Reduced response to the anti-inflammatory action of corticosteroids in stable COPD

In contrast to asthma, glucocorticoid treatment of stable COPD is rather ineffective in reducing airway inflammation and the decline of lung function.^78^ A Cochrane review of the role of regular long-term treatment with ICSs alone vs placebo in patients with stable COPD has concluded that it reduces significantly the mean rate of exacerbations and the rate of decline of quality of life but not the decline in FEV_1_ or mortality rates.^79^ Current national and international guidelines for the management of stable COPD patients recommend the use of inhaled long-acting bronchodilators, ICSs, and their combination for maintenance treatment of moderate-to-severe stable COPD.^2^ ICS treatment is also associated with adverse effects such as increased risk of oropharyngeal candidiasis, hoarseness, and pneumonia.^79^

Several large controlled clinical trials of inhaled combination therapy with ICSs and LABAs in a single device in stable COPD have shown that this combination therapy is well tolerated and produces a modest but statistically significant reduction in the number of severe exacerbations and improvement in FEV_1_, quality of life, and respiratory symptoms in stable COPD patients, with no greater risk of adverse effects than that with use of either component alone. Increased risk of pneumonia is a concern; however, this did not translate into increased exacerbations, hospitalizations, or deaths.^80^ In addition, the Towards a Revolution in COPD Health study showed a 17% relative reduction in mortality for more than 3 years for patients receiving combined salmeterol and fluticasone propionate, although this just failed to reach significance.^81^ Blood eosinophil count is a promising biomarker of the response to ICSs in COPD and could potentially be used to stratify patients for different exacerbation rate reduction strategies.^82^

Corticosteroid suppression of many inflammatory genes requires recruitment of histone deacetylases (HDACs) to the gene activation complex by the activated glucocorticoid receptor. Oxidative stress reduces HDAC2 expression and activity, thus potentially limiting glucocorticoid effectiveness in suppressing inflammation in vitro studies and in patients with COPD.^78^ Overexpression of HDAC2 restores glucocorticoid sensitivity in bronchoalveolar lavage (BAL) macrophages from COPD patients. Theophylline at concentrations that do not inhibit phosphodiesterase 4 activity enhances HDAC2 activity and functionally this enhances glucocorticoid effects.^78^ This effect may be via phosphoinositide-3-kinase (PI3K)δ-induced hyperphosphorylation of HDAC2 particularly because PI3Kδ is upregulated in peripheral lung tissue of patients with COPD.^78^ In a small clinical study of 30 patients with stable COPD treatment with low-dose slow-release theophylline plus low-dose inhaled fluticasone significantly reduced sputum neutrophils and improved lung function, and this was associated with an increase in HDAC2 activity in circulating peripheral blood monocytes.^83^ The results of an ongoing phase II controlled study (https://clinicaltrials.gov/ct2/show/NCT02130635) on the efficacy of a highly selective inhaled PI3Kδ inhibitor (GSK2269557) in patients with stable COPD are awaited with interest.

## Anticytokine and Chemokine Treatment in COPD

There is ample evidence of enhanced inflammation in COPD particularly of cytokines linked to cell recruitment and activation.^19^, ^84^, ^85^ Although many of these have been proposed to play a role in COPD pathophysiology, increased levels do not constitute evidence for a physiological role in disease, which requires efficacy in clinical trials. However, few trials of blocking antibodies against cytokines and chemokines or their receptors have proved successful. For example, although both tumor necrosis factor α (TNF-α) and CXCL8 are increased in COPD airways and sputum, the clinical effects of both anti–TNF-α and anti-CXCL8 were not encouraging.^19^ Treatment with infliximab for 6 months in a randomized, placebo-controlled trial demonstrated no clinical benefit but was associated with an increased risk of lung cancer and pneumonia.^86^ The lack of effect of anti–TNF-α in COPD is in stark contrast to the beneficial effect seen in other chronic inflammatory diseases such as rheumatoid arthritis.^87^

CXCL1, CXCL5, and CXCL8 are neutrophil chemoattractants whose expression is increased in COPD airways and who signal through CXCR2. This suggests that blocking this common receptor could reduce the chemotaxis of neutrophils.^19^ In a 6-month double-blind randomized study, patients with moderate-to-severe COPD (already on standard therapy) were given 10, 30, or 50 mg of the small molecule inhibitor of CXCR2 MK-7123 (also known as SCH527123) or placebo daily.^88^ The primary endpoint was change from baseline in postbronchodilator FEV_1_ (www.clinicaltrials.gov NCT01006616). MK-7123 resulted in a dose-dependent 67 mL improvement in FEV_1_ over placebo, a significant reduction in sputum neutrophils and reduced levels of sputum and plasma matrix metallopeptidase-9 and myeloperoxidase. The effect seen was greater in smokers compared with ex-smokers.^88^

In addition, although IL-1β amplifies inflammation and its concentration is increased in the sputum of patients with COPD, canakinumab, an anti–IL-1β-specific antibody, also had no clinical efficacy in COPD after 45 weeks of treatment (ClinicalTrials.gov identifier, NCT00581945). IL-6 concentrations increased in sputum, BAL, and serum, and because of its role in amplifying inflammation may be a useful therapeutic agent in COPD. An IL-6R–specific antibody (tocilizumab) is effective in patients with rheumatoid arthritis who are refractory to anti–TNF-α therapy^89^ but no studies have been performed in COPD patients.

Human neutrophil elastase is elastolytic, proinflammatory, and increases mucus secretion, and there is increased release from neutrophils in COPD and enhanced levels in sputum and BAL from patients with COPD. Despite AZ9668 being effective in animal models of COPD, no clinical improvement was observed for more than a period of 3 months in patients with COPD.^90^ Other proliferative agents such as epidermal growth factor whose expression is increased in COPD and enhances mucus hypersecretion have been proposed to have pathophysiological roles in COPD. Despite the effectiveness of gefitinib, for example, in some patients with lung cancer, an inhaled epidermal growth factor receptor (EGFR) inhibitor (BIBW-2948) did not substantially reduce gene expression or show any clinical benefit in patients with COPD.^91^

There has recently been much interest in the potential of targeting IL-5 in COPD patients, especially for treatment during exacerbations. Although IL-5 is associated with a Th2-driven immune response, there is evidence showing increased IL-5 levels in COPD patients during exacerbations and also in specific COPD phenotypes, including asthma-COPD overlap syndrome.^92^ It is estimated that ∼16% of COPD patients have the asthma-COPD overlap syndrome.^93^ These patients are normally younger and suffer from higher frequency and severity exacerbations compared with other COPD patients. A decrease in soluble IL-5Rα is associated with the resolution of viral-driven COPD exacerbation.^92^ Benralizumab, an anti–IL-5Rα MAb, did not reduce the rate of acute exacerbations of COPD. However, the results of prespecified subgroup analysis support further investigation of benralizumab in patients with COPD and eosinophilia.^94^ Numerous other drugs targeting specific inflammatory mediators are under investigation for COPD, and these include antibodies directed against IL-18, IL-22, IL-23, IL-33, TSLP, and Granulocyte-macrophage colony-stimulating factor (GM-CSF). It is unlikely that mouse models of COPD will be anymore predictive than those seen to date and it is only going to be possible to determine whether this approach will work is to try these approaches in COPD itself.

As seen in asthma, patient selection is likely to be critical for obtaining optimal clinical responses with anticytokine or other anti-inflammatory therapies. COPD patients present with a variable mix of distinct phenotypes such as emphysema, bronchitis, small airways disease, frequent vs infrequent exacerbators, or those with a rapid decline in lung function, which are independent of genetic background.^95^ Importantly, the level of chronic airflow obstruction is not enough to encompass the diversity of presentation of COPD.^96^ The inclusion of inflammatory markers in addition to more defined subphenotyping of COPD patients may enable the detection of COPD endotypes which, as seen with anti–IL-5Rα MAb treatment, has the potential for a more personalized treatment approach in the future as opposed to the present relatively ineffective global treatment strategy of glucocorticoids and LABA.

## Conclusions

The concept of phenotyping is not only an important step toward improved understanding of disease, but is also required to tailor asthma and COPD management to the individual patient needs as part of stratified medicine. Ultimately, phenotypes will evolve into asthma “endotypes,” which combine clinical characteristics with identifiable mechanistic pathways.

Clinical relevance of phenotyping in airways disease has been exemplified by randomized trials in asthma, for example, in which therapeutic efficacy could be predicted or improved by taking inflammatory characteristics into account. Neutrophilic airway inflammation was found to be associated with poor corticosteroid responses and corticosteroid treatment guided by eosinophilic inflammation led to better disease outcome than standard clinical management. In addition, asthma therapy with new biologicals, for example anti–IL-5 or anti–IL-13, appears to be far more effective if patients are selected using cellular (eg, eosinophils) or molecular (eg, periostin) biomarkers. This demonstrates the key role of individual inflammatory mediators in driving the inflammatory response in asthma and highlights the need for accurate disease (sub)phenotyping to optimize disease management and drug development.

The use of novel inflammatory mediator-targeted therapeutic agents in selected patients with asthma or COPD and the detection of markers of responsiveness or nonresponsiveness will allow a link between clinical phenotypes and pathophysiological mechanisms to be delineated reaching the goal of endotyping each patient. Early blood or exhaled biomarkers of subphenotypes and/or treatment response will be important to ensure that patients can be taken off a treatment early if it is ineffective to reduce the risk of any possible adverse effects in an adaptive design clinical study or in real life.
